# HRIBO: high-throughput analysis of bacterial ribosome profiling data

**DOI:** 10.1093/bioinformatics/btaa959

**Published:** 2020-11-11

**Authors:** Rick Gelhausen, Sarah L Svensson, Kathrin Froschauer, Florian Heyl, Lydia Hadjeras, Cynthia M Sharma, Florian Eggenhofer, Rolf Backofen

**Affiliations:** Bioinformatics Group, Department of Computer Science, University of Freiburg, 79110 Freiburg, Germany; Chair of Molecular Infection Biology II, Institute of Molecular Infection Biology (IMIB), University of Würzburg, 97080 Würzburg, Germany; Chair of Molecular Infection Biology II, Institute of Molecular Infection Biology (IMIB), University of Würzburg, 97080 Würzburg, Germany; Bioinformatics Group, Department of Computer Science, University of Freiburg, 79110 Freiburg, Germany; Chair of Molecular Infection Biology II, Institute of Molecular Infection Biology (IMIB), University of Würzburg, 97080 Würzburg, Germany; Chair of Molecular Infection Biology II, Institute of Molecular Infection Biology (IMIB), University of Würzburg, 97080 Würzburg, Germany; Bioinformatics Group, Department of Computer Science, University of Freiburg, 79110 Freiburg, Germany; Bioinformatics Group, Department of Computer Science, University of Freiburg, 79110 Freiburg, Germany; Signalling Research Centres BIOSS and CIBSS, University of Freiburg, 79104 Freiburg, Germany

## Abstract

**Motivation:**

Ribosome profiling (Ribo-seq) is a powerful approach based on deep sequencing of cDNA libraries generated from ribosome-protected RNA fragments to explore the translatome of a cell, and is especially useful for the detection of small proteins (50–100 amino acids) that are recalcitrant to many standard biochemical and *in silico* approaches. While pipelines are available to analyze Ribo-seq data, none are designed explicitly for the automatic processing and analysis of data from bacteria, nor are they focused on the discovery of unannotated open reading frames (ORFs).

**Results:**

We present HRIBO (High-throughput annotation by Ribo-seq), a workflow to enable reproducible and high-throughput analysis of bacterial Ribo-seq data. The workflow performs all required pre-processing and quality control steps. Importantly, HRIBO outputs annotation-independent ORF predictions based on two complementary bacteria-focused tools, and integrates them with additional feature information and expression values. This facilitates the rapid and high-confidence discovery of novel ORFs and their prioritization for functional characterization.

**Availability and implementation:**

HRIBO is a free and open source project available under the GPL-3 license at: https://github.com/RickGelhausen/HRIBO.

## 1 Introduction

**Fig. 1.**
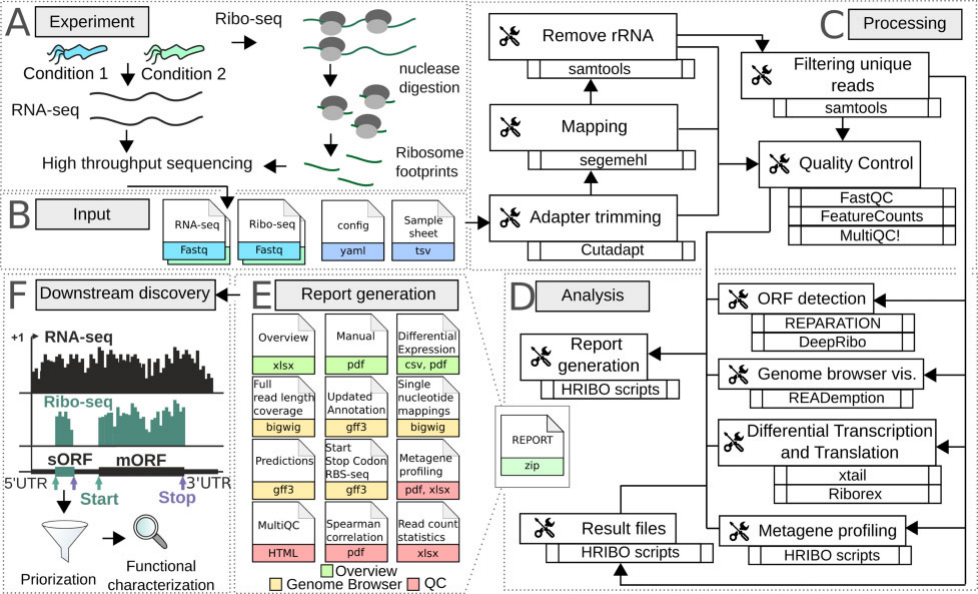
Bacterial Ribo-seq data analysis by the HRIBO pipeline. (**A**) In Ribo-seq, parallel RNA-seq and ribosome footprint libraries are prepared from bacterial cultures, optionally from multiple experimental conditions and replicates, and deep sequenced. (**B**) The fastq files from each library, config file and a sample sheet defining the experimental setup serve as input for HRIBO, which automatically downloads software dependencies and executes commands for processing (**C**), which ensures reproducibility. For all the processing steps quality control measures are computed and summarized by MultiQC. (**D**) First, coverage files for Genome browser visualization are generated, including three variants of single nucleotide mapping, and global metagene profiling of ribosome occupancy near start codons is calculated. Next, two ORF prediction tools (REPARATION and DeepRibo) detect translated annotated and novel ORFs from the ribosome footprint libraries. The detected ORFs are then screened together for differential transcription and translation. Finally, the expression information for each ORF is then aggregated together with additional features (nucleotide sequence, length, etc.) in an overview results table. (**E**) Files from all analysis steps are bundled together, along with a manual, into a report archive. (**F**) Overall, HRIBO streamlines the selection of high-confidence, functionally important, translated novel ORFs for further experimental investigation. For more details please refer to the documentation: https://hribo.readthedocs.io/en/latest

Ribosome profiling (Ribo-seq) (Ingolia et al., 2009) is an RNA-seq based approach that identifies the ribosome-bound fraction of the transcriptome as a proxy for protein expression (Fig. 1A). Because RNase digestion of mRNA regions not protected by translating ribosomes creates so-called ribosome footprints, it also allows definition of open-reading frame (ORF) boundaries. This makes it remarkably suited to detect small proteins, which are currently underrepresented in genome annotations due to length cutoffs imposed during annotation, as well as unique features that preclude their detection by conventional experimental or *in silico* approaches (Storz et al., 2014). In addition, a parallel whole-transcriptome library allows calculation of translation efficiency (TE, the ratio of footprint library coverage to transcriptome coverage) and identification of ORFs that might be differentially expressed under conditions relevant to the organism studied, such as those encountered during infection. There are existing workflows for the analysis of eukaryotic Ribo-seq data (Chung et al., 2015; Wang et al., 2019), but a dedicated, automatic solution for bacteria is still missing. Here, we present HRIBO (High-throughput annotation by Ribo-seq), a computational pipeline that processes and analyses data from any bacterial Ribo-seq experiment, but also detects translated novel ORFs. The tool is compatible with bacterial annotations and circular chromosomes, uses an optimized mapping approach suitable for small bacterial genomes, integrates machine learning-based ORF prediction tools designed for/trained on bacterial ORF features, and also includes two differential expression tools designed for Ribo-seq data. We implemented HRIBO based on snakemake (Köster et al., 2012), which allows highly reproducible and fully automatic data analysis.

## 2 Approach

HRIBO automatically retrieves tools from bioconda (Grüning *et al.*, 2018) and performs all necessary steps of processing, with pinned and tested tool versions. Detailed documentation, with examples, is available at: https://hribo.readthedocs.io/en/latest/.

HRIBO (Fig. 1B) requires the sequencing data files (paired RNA-seq and Ribo-seq libraries for each sample), the genomic sequence/annotation of the organism and a sample sheet that captures the experimental setup by associating samples/replicates with their experimental condition. As a first step (Fig. 1C), HRIBO performs adapter trimming with Cutadapt (Martin, 2011) and then maps the reads to the genome with segemehl, which has higher sensitivity than other mappers (Otto et al., 2014), but its high computational costs are still acceptable for small genomes. Multimapping/rRNA reads are then removed with samtools (Li *et al.*, 2009) before further processing. FastQC (Andrews et al., 2010) and featurecount (Liao et al., 2014) reports are created after each processing step and aggregated with MultiQC (Ewels et al., 2016), enabling the investigator to identify problems with either the experimental setup (e.g. insufficient rRNA depletion), or the pre-processing (e.g. untrimmed adapters). The resulting mapped reads are then used in subsequent steps (Fig. 1D) to calculate read statistics, detect expressed ORFs (annotated or novel), compute differential expression, generate coverage tracks and perform metagene profiling of global ribosome density near start codons. Notably, we have included two complementary ORF detection tools, both specifically developed for bacterial organisms. REPARATION (Ndah et al., 2017) uses a random forest-based machine-learning approach. DeepRibo (Clauwaert et al., 2019) is based on convolutional and recurrent neural network approaches. The results of the two prediction tools are aggregated by newly developed scripts and compiled into a list of all detected ORFs (both annotated and novel). ORFs that are differentially expressed (both transcription and translation) between samples/conditions are detected using the complementary Ribo-seq-specific tools xtail (Zhang et al., 2017) and Riborex (Li et al., 2017). The list is then enriched with additional computed context and expression information for each ORF, such as length, sequence, normalized read counts, TE, available annotation/novelty, differential expression, and which conditions/replicates the ORF was detected in. Interesting candidates can then be inspected by either full read coverage (Förstner et al., 2014) or single nucleotide mapping (5′/3′ end or center of read) genome browser tracks. Moreover, we developed metagene profiling scripts that globally analyze read density around start codons, including for different read lengths. This allows the inspection of data quality, examination for organism-specific ORF/translatome signatures and potentially the identification of optimal read lengths that could be used to detect three-nucleotide periodicity in ORFs. Finally, the results are collected into a report (Fig. 1E) that can be easily distributed and contains a detailed manual. HRIBO allows priorization and identification of novel ORFs (see Fig. 1F), e.g. sORFs in *Salmonella Typhimurium* related to virulence (Venturini et al., 2020). An example HRIBO report for a published dataset (Potts et al., 2019), which required 4 h 4 min on 12 cores of an AMD EPYC CPU (@ 1996 MHz) to compute, can be found here: ftp://biftp.informatik.uni-freiburg.de/pub/HRIBO/HRIBO1.4.4_18-09-20.zip.

## 3 Conclusion

HRIBO is a reproducible and standardized pipeline that includes all tools required to process Ribo-seq datasets from bacterial organisms, from pre-processing and quality control to ORF prediction and differential expression analysis. Read information, differential transcription/translation and additional computed features for both annotated and predicted ORFs are summarized in a single table that can be easily inspected together with the generated genome browser tracks. This streamlines the selection of high confidence, functionally important, translated novel ORFs for further experimental investigation. Existing pipelines are, other than the exceptions discussed below (Choe et al., 2020; Fremin et al., 2020; Weaver et al., 2019), not specifically developed for bacterial organisms. Most existing pipelines do not cover the complete analysis workflow and lack the initial processing steps, including mapping (see Fig. 1 of Ribominer) (Li et al., 2020). Moreover, the complete workflows frequently use mapping tools with a lower runtime, at the cost of lower sensitivity, to allow processing of large eukaryotic genomes in an acceptable time-scale (Michel et al., 2016), or they support only a small set of bacterial organisms (Verbruggen et al., 2019). Furthermore, none of the pipelines feature ORF discovery tools specialized for bacteria. This is not only of importance due to decreased sensitivity for bacterial translation signatures, but also because some tools are not compatible with circular bacterial genomes, meaning that ORFs that span the origin might have negative coordinates. In addition, the different architecture of bacterial annotations, which include operons instead of introns and exons, also preclude their use with some eukaryotic tools (Calviello et al., 2016). The three pipelines and protocols also developed for bacterial organisms are either built for different objectives, or they offer other functionality than HRIBO. The pipeline used to process Ribo-Seq and translation initiation site profiling data in bacteria (Weaver *et al.*, 2019), as well as in archaea (Gelsinger et al., 2020) consists of a collection of semi-automatic ipython notebooks which perform the pre-processing of the data. In addition, while the pipeline performs some post-processing calculations (such as pause score and gene density analysis), it does not include bacterial ORF prediction tools that work solely on Ribo-seq (rather than initiation site profiling) coverage files. Moreover, it does not perform the additional analysis steps on predicted ORFs performed by HRIBO such as calculation of translation efficiency, differential expression analysis and computation of additional ORF features. MetaRiboSeq (Fremin *et al.*, 2020) is an experimental and computational protocol to investigate the transitional landscape of a metagenomic sample. However, it considers only proteins that satisfy a specific RPKM threshold and are also considered homologous to proteins in a database as translated, while HRIBO, which has been designed for use with a single organism, allows the use of more sophisticated and computationally more costly tools. Furthermore, the MetaRiboSeq computational approach is only described in the manuscript and not implemented as a script or tool, therefore it does not offer any automation. STATR (Choe *et al.*, 2020), in contrast to HRIBO, is a semi-automatic analysis protocol, requiring the manual installation of all software dependencies and execution of tool commands and does not, unlike HRIBO, offer quantification and removal of rRNA and multi-mapping reads, sequencing quality control reports, ORF detection/prediction, differential transcription and translation output and customized genome browser tracks. Recently, translation initiation site profiling, where ribosomes are enriched at start codons by treatment with antibiotics that specifically target initiating ribosomes (Gelsinger *et al.*, 2020; Meydan et al., 2019; Weaver *et al.*, 2019), has now been adapted for bacteria and archaea. Therefore, as an update to HRIBO, we plan to add a module allowing the reproducible analysis of translation initiation site profiling data.
