# Corrigendum to: HRIBO: high-throughput analysis of bacterial ribosome profiling data

**DOI:** 10.1093/bioinformatics/btab256

**Published:** 2021-07-16

Rick Gelhausen, Sarah L. Svensson, Kathrin Froschauer, Florian Heyl, Lydia Hadjeras, Cynthia M. Sharma, Florian Eggenhofer and Rolf Backofen

*Bioinformatics* (2020) doi: 10.1093/bioinformatics/btaa959

Upon the original publication of this article, the following funding source was erroneously omitted from the Funding section: “DFG, Germany's Excellence Strategy – EXC-2189 – Project ID: 390939984”. This error has been corrected.

